# DICOM re‐encoding of volumetrically annotated Lung Imaging Database Consortium (LIDC) nodules

**DOI:** 10.1002/mp.14445

**Published:** 2020-09-06

**Authors:** Andrey Fedorov, Matthew Hancock, David Clunie, Mathias Brochhausen, Jonathan Bona, Justin Kirby, John Freymann, Steve Pieper, Hugo J. W. L. Aerts, Ron Kikinis, Fred Prior

**Affiliations:** ^1^ Brigham and Women’s Hospital Boston MA 02115 USA; ^2^ Florida State University Tallahassee FL 32306 USA; ^3^ PixelMed Publishing Bangor PA 18013 USA; ^4^ University of Florida Health Gainesville FL 32605 USA; ^5^ University of Arkansas for Medical Sciences Little Rock AR 72205 USA; ^6^ Frederick National Laboratory for Cancer Research Frederick MD 21701 USA; ^7^ Isomics Inc. Cambridge MA 02138 USA

**Keywords:** cancer imaging, DICOM, FAIR data, image annotations, lung cancer, quantitative imaging

## Abstract

**Purpose:**

The dataset contains annotations for lung nodules collected by the Lung Imaging Data Consortium and Image Database Resource Initiative (LIDC) stored as standard DICOM objects. The annotations accompany a collection of computed tomography (CT) scans for over 1000 subjects annotated by multiple expert readers, and correspond to “nodules ≥ 3 mm”, defined as any lesion considered to be a nodule with greatest in‐plane dimension in the range 3–30 mm regardless of presumed histology. The present dataset aims to simplify reuse of the data with the readily available tools, and is targeted towards researchers interested in the analysis of lung CT images.

**Acquisition and validation methods:**

Open source tools were utilized to parse the project‐specific XML representation of LIDC‐IDRI annotations and save the result as standard DICOM objects. Validation procedures focused on establishing compliance of the resulting objects with the standard, consistency of the data between the DICOM and project‐specific representation, and evaluating interoperability with the existing tools.

**Data format and usage notes:**

The dataset utilizes DICOM Segmentation objects for storing annotations of the lung nodules, and DICOM Structured Reporting objects for communicating qualitative evaluations (nine attributes) and quantitative measurements (three attributes) associated with the nodules. The total of 875 subjects contain 6859 nodule annotations. Clustering of the neighboring annotations resulted in 2651 distinct nodules. The data are available in TCIA at https://doi.org/10.7937/TCIA.2018.h7umfurq.

**Potential applications:**

The standardized dataset maintains the content of the original contribution of the LIDC‐IDRI consortium, and should be helpful in developing automated tools for characterization of lung lesions and image phenotyping. In addition to those properties, the representation of the present dataset makes it more FAIR (Findable, Accessible, Interoperable, Reusable) for the research community, and enables its integration with other standardized data collections.

## 1 BACKGROUND AND SUMMARY

It is estimated that medical imaging generates millions of clinical scans annually in the US alone.^1^ Few of those scans become available for training of Artificial Intelligence (AI) systems, and even fewer are accompanied by annotations (labels localizing imaging findings and structured metadata describing various aspects of the disease and the imaged subject). Lack of such curated datasets has been acknowledged as a major bottleneck, if not the biggest challenge in the field of deep learning as applied to medical imaging.^1^, ^2^ Curated and annotated medical imaging collections do exist, however. A prominent example of such collection is the one generated by the Lung Imaging Database Consortium and Image Database Resource Initiative (LIDC/IDRI, further referred to as LIDC), which has been a major effort supported by the National Cancer Institute (NCI) to establish a publicly available reference database of computed tomography (CT) images for detection, classification and quantitative assessment of lung nodules.^3^, ^4^, ^5^ In an effort spanning multiple years, LIDC collaboration involved seven academic centers and eight medical imaging companies to collect a multi‐site collection of CT scans for over 1000 subjects where each case was annotated by up to four experienced thoracic radiologists from the LIDC reader pool to both localize and characterize identified nodules. The resulting collection consists of the CT images stored using Digital Imaging and Communications in Medicine (DICOM) format and annotations in an XML format that follows a project‐specific schema.^6^ The latter was done for convenience during the data collection process, and (at the time) in absence of any existing standard format or best practices for sharing image annotations. To increase visibility and facilitate access to the resulting collection, it has been published using the resources of The Cancer Imaging Archive (TCIA).^7^

The choice of representation for storing the resulting annotations was developed for convenience during the data collection process, and in absence of existing standard format and best practices for sharing such annotations. It proved to be effective when implemented to support radiologists annotating the data using custom software tools designed specifically for the project. Reuse of those XML annotations outside of the Consortium is more complicated. No publicly available tools were provided to accompany the dataset to either consume the annotations and support their visualization, or to provide conversion of the contours into formats supported by the platforms commonly used by imaging researchers. Our experience shows that errors in implementing conversion of XML contours into alternative representation are not uncommon (e.g., we are aware of implementations that erroneously include contour points into the nodule segmentation volume). The representation is not self‐contained, requiring the consumer of the annotations to carefully examine accompanying documentation to understand the conventions used in labeling of the nodules and the meaning of codes used for nodule characterization. This project‐specific XML format makes it challenging to harmonize the annotations with the other similar artifacts (imaging data, annotations and analysis results generated for other projects) within TCIA to support search and query of the data.

Despite all the challenges mentioned above, the dataset has proven to be of high value, and has become one of the most popular TCIA collections. The publications describing the dataset^3^, ^4^, ^5^ have accumulated over 1000 citations according to Google Scholar, and have been used in a number of image analysis challenges.^8^ Several tools (some of which have been released publicly) have been contributed by the community to enable conversion of the XML annotations into alternative representations and to support exploration of the collection content.^9^, ^10^, ^11^, ^12^, ^13^, ^14^ Nevertheless, until very recently, the XML annotations have been the only representation accessible to the users of the TCIA LIDC‐IDRI collection.

This manuscript presents a standardized DICOM representation of the annotations corresponding to the volumetrically annotated nodules ≥3 mm produced by the LIDC project. Although the project also produced annotations of non‐nodules ≥3 mm and nodules <3 mm, those were not included in the present effort. There are several advantages of the DICOM representation as compared to a project‐specific format. As we demonstrate in this manuscript, such a representation makes it possible to use the dataset with generic existing tools that support relevant components of the DICOM standard and were not developed specifically for this dataset — something not previously possible. The DICOM representation better satisfies FAIR (Findable Accessible Interoperable Reusable) guiding principles for scientific data management and stewardship.^15^ This approach also enables harmonization of the annotations of this specific dataset with conceptually similar results of analysis available for other collections of TCIA. As a result, aggregated queries across collections and across data types become possible. It also becomes easier to extend the dataset with new types of data. As an example, the same mechanisms for data encoding could be used for augmentation of the images and nodule annotations with the radiomics features^16^ derived from the nodule regions. Finally, it becomes straightforward to use existing tools that operate on DICOM data to visualize and reuse the resulting objects. This work makes use of tools developed earlier for interpreting XML annotations of LIDC^13^ and for generating the standardized DICOM representations for image analysis results.^17^, ^18^ A preprint describing this approach and dataset appeared earlier.^19^

## 2 MATERIALS AND METHODS

### 2.A Introduction of the overall approach

We describe the process that was used to generate DICOM objects that contain annotations of lung nodules, their qualitative evaluations and numeric measurements. Originally, this image‐derived data was stored in XML files accompanying the DICOM CT images.^5^ A “pilot release” containing 399 subjects of the LIDC CT data was published in 2009. In June 2011 the final data set was published on TCIA including all 399 pilot CT cases plus the additional 611 patient CTs and 290 corresponding chest x‐rays. The data have been de‐identified and curated per standard operating procedures of TCIA^7^, ^24^ to ensure no identifiable subject information is included. Both DICOM CT images and XML files are publicly shared in the TCIA LIDC‐IDRI collection.^6^ The DICOM dataset presented in this manuscript and containing the DICOM encoded annotations is available on TCIA at https://doi.org/10.7937/TCIA.2018.h7umfurq.^20^

An understanding of the content of XML annotations produced by the LIDC initiative can be gained through the peer‐reviewed manuscripts published by the initiative,^3^, ^4^, ^5^ and the documentation linked from the TCIA LIDC‐IDRI collection page.^6^ Briefly, the initiative distinguished between the three groups of findings, as defined by Armato et al.^5^: “(a) “nodules ≥ 3 mm” (defined as any lesion considered to be a nodule with greatest in‐plane dimension in the range 3–30 mm regardless of presumed histology); (b) “nodules < 3 mm” (defined as any lesion considered to be a nodule with greatest in‐plane dimension <3 mm that is not clearly benign); and (c) “non‐nodules ≥ 3 mm” any other pulmonary lesion, such as an apical scar, with greatest in‐plane dimension greater than or equal to 3 mm that does not possess features consistent with those of a nodule)”. Each of up to four radiologists assigned to read each case independently reviewed all of the scans in a “blinded” phase to identify all of the findings from the three groups above. For each finding identified by a given radiologist as a *“*nodule ≥ 3 mm*”*, outlines were constructed in each slice where that nodule appeared; for the other two categories only the approximate center of mass was annotated. In the subsequent “unblinded” read phase each radiologist had access to the categories assigned and annotations for the nodules, and “a radiologist’s own marks then could be left unchanged, deleted, switched in terms of lesion category, or additional marks could be added.”^5^ After the unblinded phase, each radiologist assessed subjective characteristics of “nodules ≥ 3 mm,” such as spiculation, subtlety, etc (as discussed further).

We limited the scope of our conversion to include only “nodules ≥ 3 mm.” For those nodules, the XML annotations contain the following:

1. Planar contours that define “included” or “excluded” regions of a nodule in a given image from the CT series, organized in groups corresponding to the individual nodules. These contours are defined as a list of image‐relative coordinates, and correspond to the pixels just outside the nodule (i.e., the contour pixels themselves should not be treated as belonging to the nodule).
2. Attributes describing various characteristics of the nodule such as opacity, conspicuity, etc. using project‐specific codes.

Our approach to re‐encoding this data in a standard form uses existing DICOM object definitions. A DICOM Segmentation object (SEG)^21^ is the standard way to encode segmentations defined as labeled image voxels. DICOM Structured Reporting (SR)^22^ provides a versatile mechanism for communicating image‐based measurements, and supports both quantitative and qualitative evaluations using template TID 1500 (SR‐TID1500).^23^

Compared to a project‐specific XML representation, DICOM representation offers the following advantages (also described elsewhere^17^):

● Every DICOM object is uniquely identified by a SOPInstanceUID, and is suitable for storage side by side with the DICOM CT dataset, so the resulting content can be archived, queried and retrieved using standard DICOM storage capabilities.
● Attributes of the composite context (patient identification and descriptive attributes such as gender and age, and unique identifiers for the study) are included directly in the object in the standard data elements using well‐defined encoding rules.
● The use of generic, standard DICOM objects increases the possibility of using this data with general‐purpose tools. A number of open source and commercial tools already include support for both SEG and SR‐TID1500.
● There are standard representations for DICOM content converted into XML or JSON.
● There are tools for automatic validation that the DICOM objects conform to the standard.

DICOM SEG objects have a number of desirable features for encoding segmentations. SEG objects belong to the family of DICOM enhanced multiframe objects, which means that all of the slices of the segmentation are stored in a single instance. The semantics of the segmentation are encoded in standard data elements, and for values, use standard codes from existing terminologies. References to the images being segmented can be included directly in the SEG, making it easier to trace the provenance of the object and for visualization tools to automatically retrieve the image corresponding to the segmentation. There are standard data elements to describe recommended colors for visualization of segments superimposed on images, which is particularly helpful when multiple segments for a single finding are available, as is the case in the TCIA LIDC‐IDRI collection.

DICOM SR^22^ uses data elements to encode a higher level abstraction that is a tree of content, where nodes of the tree and their relationships are formalized. SR‐TID1500 is one of many standard templates that define constraints on the structure of the tree, and is intended for generic tasks involving image‐based measurements. DICOM SR uses standard terminologies and codes to deliver structured content. These codes are used both for defining the concept names and values assigned to those concepts (name‐value pairs). Measurements include coded concepts corresponding to the quantity being measured, and a numeric value accompanied by coded units. Coded categorical or qualitative values may also be present. In SR‐TID1500, measurements are accompanied by additional context that helps interpret and reuse that measurement, such as finding type, location, method and derivation. Measurements computed from segmentations can reference the segmentation defining the region and the image segmented, using unique identifiers of the respective objects.

The conversion process was implemented as a python script parameterizing and executing individual converters as needed. The source code of the conversion script, accompanying Jupyter Notebook, and other related items are available at https://github.com/QIICR/lidc2dicom.

### 2.B Encoding of nodule annotations

Our approach to creating DICOM SEG representation of the nodule outlines was to use existing tools to enable the conversion process.

First, this work leveraged the *pylidc* python package (https://pylidc.github.io/) introduced by Hancock and Magnan^13^ for accessing the volume‐reconstructed annotation contours for the individual scans and subjects, as extracted from the DICOM and XML components of the TCIA LIDC‐IDRI collection. *pylidc* provides an interface for iterating and querying various entities of the collection and their attributes. It also reconstructs filled multi‐slice segmentations from the per‐slice annotation contours. The resulting segmentations are represented as three‐dimensional *numpy* arrays, which lack the geometry information needed to align them with the reference frame of the CT image. We reoriented and augmented the *numpy* representation with the resolution and geometric position to construct a fully defined volume in the frame of reference of the source CT series. We utilized the Plastimatch software^25^ (http://plastimatch.org/) for generating volume reconstructions of the CT scans, which was then used to define the geometry of the segmentation.

**Fig. 1:**
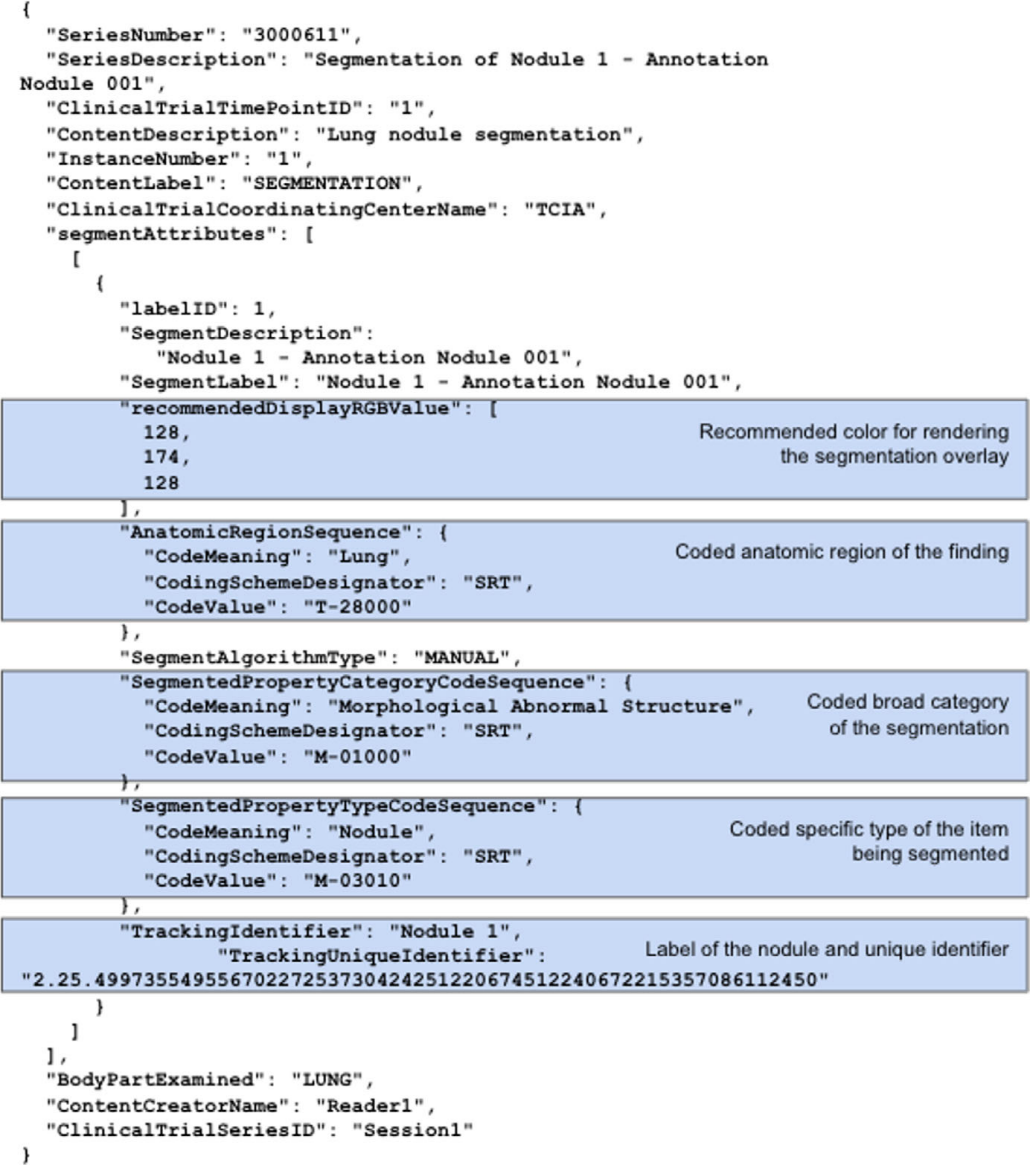
Example JSON file used to parameterize conversion of a nodule annotation into DICOM SEG representation. Coded items are defined as triplets of (CodeMeaning, CodingSchemeDesignator, CodeValue), where “SRT” denotes the SNOMED RT Identifier used to refer to SNOMED‐CT concepts. [Color figure can be viewed at wileyonlinelibrary.com]

The resulting volume, now fully defined in the frame of reference of the CT image, was then exported into NRRD format using ITK python package (https://itkpythonpackage.readthedocs.io/), and then the *itkimage2segimage* tool from the *dcmqi* library^18^ (https://github.com/qiicr/dcmqi) was used to generate a standard DICOM SEG object. The DICOM SEG conversion was parameterized by two sets of information. First, metadata from the source CT instances was used to propagate composite context information and to provide the identifiers for references to the source images in the result. This step is performed automatically by the converter, given the pointer to the CT series being segmented. Second, metadata describing the segmentation was populated using a schema‐constrained JSON file. An example of such a file is shown in Fig. 1. Semantics of the segmentation were defined by the SNOMED RT (SRT) identifiers that refer to SNOMED‐CT codes. At the time this work was performed, DICOM used SRT identifiers rather than SCT numeric codes to specify SNOMED‐CT concepts, prior to the adoption of CP 1850. The TCIA dataset has not been updated to replace the SRT codes with SCT codes for the same concepts. The Segmentation Category value was chosen from the list of codes defined in DICOM context group CID 7150 “Segmentation property category,” and always set to “Morphologically altered structure” (SNOMED Concept ID (SCTID) 49755003). The Segmentation Type was chosen from CID 7151 “Segmentation property type”, and always set to “Nodule” (SCTID 27925004). The Anatomic Region was selected from DICOM CID 4 “Anatomic Region”, and always set to “Lung” (SCTID 39607008). DICOM context groups are defined in Section B of Part 16 of the standard.^26^

**Fig. 2:**
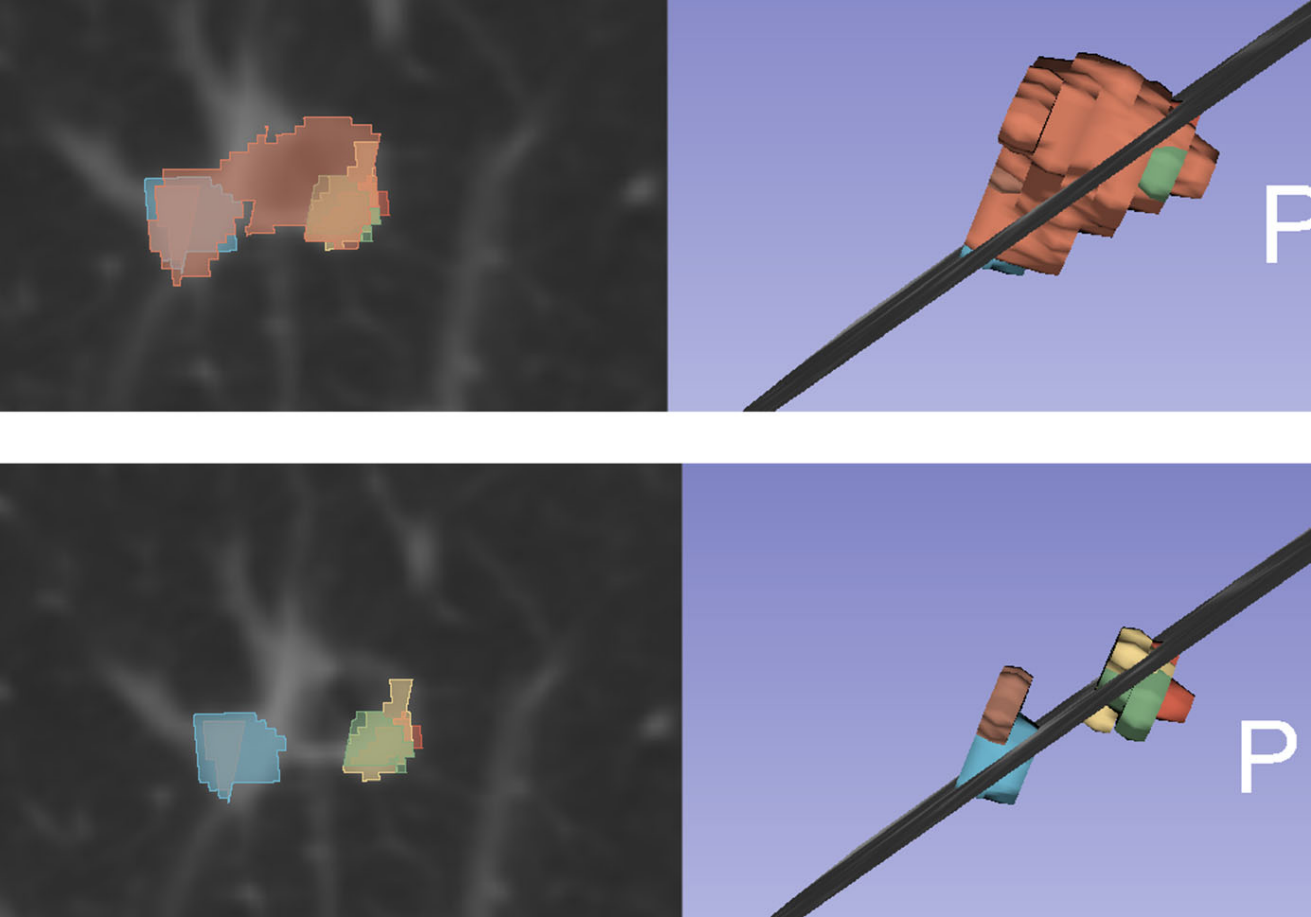
Illustration of annotations for subject LIDC‐IDRI‐0055 where in one instance (top) nodule was segmented as a single continuous structure, while other sets of annotations appear to segment separate components of the nodule (bottom). Lacking nodule or reader identifiers in the original LIDC/IDRI XML annotations, it is not clear how to ascertain whether annotations shown in the bottom figure correspond to two separate nodules, or to a single nodule. All of the annotations were assigned to the same cluster by pylidc and were encoded as belonging to the same nodule in the DICOM SEG representation. [Color figure can be viewed at wileyonlinelibrary.com]

Most of the nodules were annotated by more than one expert reader. Annotations, as generated by the consortium, do not contain metadata indicating whether those individual annotations correspond to the same physical nodule. We utilized automatic clustering of the annotations into groups by computing a distance measure between the annotations, and assigning the annotations in the resulting clusters to have the same nodule label (see details on automatic clustering in Ref. [27]). Note that in 14 nodules the total number of automatically clustered annotations is above 4 (the maximum number of radiologists interpreting any given nodule). In those cases some nodules may be too close and must be grouped manually. Assignment of an annotation to a given nodule is reflected in the SeriesDescription, SegmentDescription, SegmentLabel, TrackingID (human‐readable label unique within a given scan) and TrackingUID (unique identifier across all subjects) attributes. TrackingID and TrackingUID are maintained between SEG and SR‐TID1500 instances. In addition, each of the annotations that were clustered to the same nodule are assigned identical and unique TrackingUniqueIdentifier values. The identity of the reader was intentionally not exposed by the LIDC initiative. As such, it is impossible to ascertain whether any two annotations were performed by the same reader. Furthermore, some of the nodules could be interpreted as having multiple components by one reader, but might have been annotated as a single nodule by another reader (e.g., see Fig. 2).

The following decisions were made and these conventions were followed:

● **Only nodules that were contoured volumetrically are considered.** That is, nodules that were less than the threshold used in the LIDC study, or which had only the center identified, were not processed.
● **Each individual annotation of a nodule is saved as a separate DICOM segmentation object.** Visualization of the segmentations might be more convenient had all segmentations of a nodule been stored in a single DICOM object. However, reader identification was not provided for the individual annotations, and we decided not to group content potentially generated by different readers into a single DICOM object.
● **Segmentation object SeriesDescription (and matching SegmentDescription and SegmentLabel attributes) follow the convention “Nodule < nodule number> — Annotation < annotation ID>”.** Nodule number is a consecutive number assigned as provided by *pylidc*, which uses spatial clustering of the individual annotations for a given scan to associate those to the same nodule. <annotation ID> is the identifier as assigned to the individual annotations in the XML annotation files.
● **Each nodule is assigned a unique tracking identifier, which is stored in the DICOM TrackingUID attribute per segment.** This allows annotations corresponding to the same nodule to be linked. The same tracking identifier is used in the DICOM SR TID1500 Measurement group containing the qualitative assessments and measurements (in addition to explicit references to the UID and Segment Number of the corresponding SEG).
● **Segmentation display colors.** To make visualization of the nodule segmentations more user‐friendly, individual annotations for a given nodule were assigned distinct, prominent colors in the RecommendedDisplayCIELabValue attribute. No implications about the relationship among any of the annotations that use the same colors (e.g., that they were done by the same reader) should be made: the color is used purely to facilitate visualization of the annotations superimposed on the images.
● **Empty frames were not included in the DICOM Segmentation objects.** The spatial extent of the pixel data of the encoded Segmentation objects is less than the entire volume spanned by the source CT images, in that slices above and below the nodules have been omitted. This reduces the size of the encoded data. A further optimization, constraining the size of the encoded volume to a cuboid bounding box around the nodule, though permitted by the DICOM standard, was not performed.

### 2.C Encoding of annotation‐derived characterizations and measurements

In the LIDC protocol, all of the “nodules ≥ 3 mm” were subjectively assessed to describe characteristics of the nodule such as subtlety, internal structure, spiculation, lobulation, shape, sphericity, solidity, margin, and likelihood of malignancy.^4^, ^5^ For each of those characteristics, a numeric score or category was assigned, and stored in the LIDC XML representation. Explanation of the meaning of those scores or categories was provided in a separate explanatory document accompanying the XML annotations, and available on the TCIA LIDC‐IDRI collection page.^6^ Lack of self‐contained description of the score meaning creates at least a potential for accidental reversal of the ratings.

In order to generate DICOM SR‐TID1500 representation of those characterizations, it was necessary to select codes corresponding to the concepts and values used in the original annotation process. The original LIDC effort did not make use of standard codes. Therefore, we first made an attempt to identify codes in existing terminologies and ontologies corresponding to the project‐specific concepts and values assigned to those concepts. This review included NCI Thesaurus,^28^ RadLex™^29^ and the subset of Systematized Nomenclature of Medicine (SNOMED^®^)^30^ codes included in the DICOM standard. To identify matching codes, we used BioPortal^31^ (http://bioportal.bioontology.org/), the Ontology Lookup Service^32^ (https://www.ebi.ac.uk/ols), and the RadLex Term Browser (http://www.radlex.org/). We also considered the terms defined by the Imaging Biomarker Standardization Initiative (IBSI),^16^ which defines concepts in the context of radiomics feature extraction. We also consulted a report by Opulencia et al.^33^ mapping LIDC concepts to RadLex and refined our selection accordingly. Where matches were identified, standard codes were used. However, if the match was deemed to have the potential of losing the project‐specific meaning, we opted for introducing our own private (nonstandard) codes. The nonstandard new codes are identified by the “99LIDCQIICR” coding scheme (where the prefix “99” is the DICOM mechanism for flagging a coding scheme as nonstandard). The codes used are summarized in Appendix S1.

In addition to the subjective characterizations, we included the measurements calculated by *pylidc,* coded as follows:

● Diameter: (“M‐02550”, “SRT”, “Diameter”), units: (“mm”, “UCUM”, “millimeter”)
● Surface area: (“C0JK”, “IBSI”, “Surface area of mesh”), units: (“mm2”, “UCUM”, “square millimeter”)
● Volume: (“G‐D705”, “SRT”, “Volume”), units: (“mm3”, “UCUM”, “cubic millimeter”)

Qualitative characterizations were extracted from XML and associated with the nodule annotations using *pylidc*. Generation of DICOM SR‐TID1500 content was done using the *tid1500writer* tool from *dcmqi*. Similar to the process of generating DICOM SEG, the conversion process was parameterized using our own nonstandard schema‐constrained JSON, which described the characterizations and measurements to be encoded, and associated them with the segmentations and source CT images that were used to derive them.

### 2.D Validation

Conformance of the converted objects to the DICOM standard was established using the *dciodvfy* tool from the *dicom3tools* software^34^ (http://www.dclunie.com/dicom3tools.html). Consistency checks were performed between the annotations and metadata stored in the DICOM objects and the *pylidc* database. Those consistency checks were performed by extracting DICOM metadata from the SEG and SR‐TID1500 objects using the Google Healthcare tools (as further discussed in the Usage section), and utilizing a combination of Google BigQuery queries and procedures implemented using Google Colab notebooks. Specifically, we confirmed that

1. every clustered annotation nodule and individual annotation of the lesion in *pylidc* has a corresponding DICOM SEG object;
2. the concepts and value sets used for coding qualitative and quantitative evaluations are consistent with what is presented in Appendix S1.

**Fig. 3:**
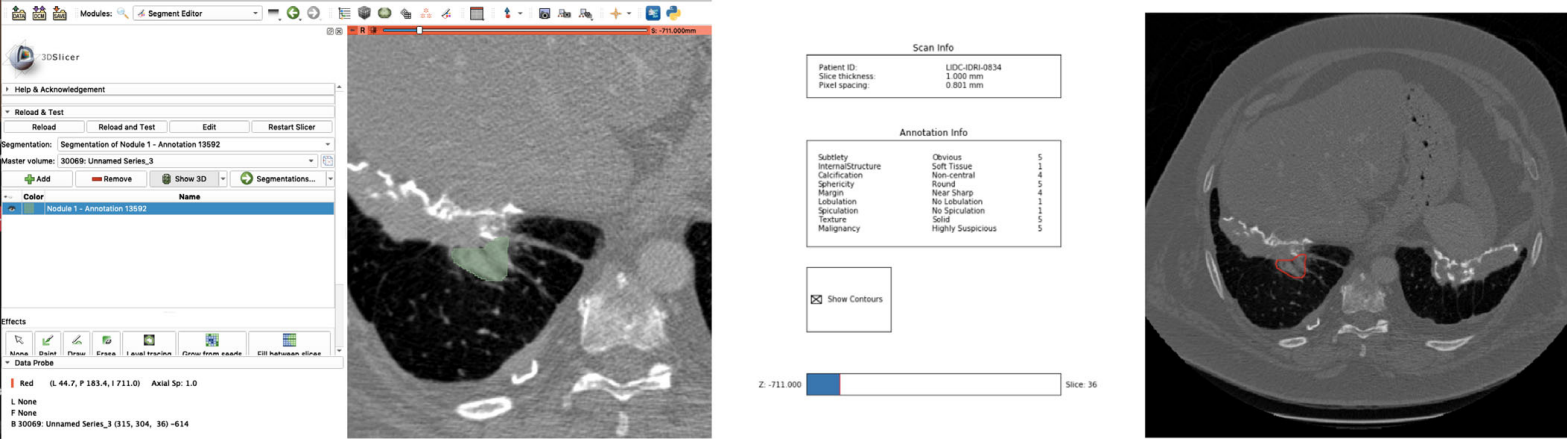
Visualization of the same nodule annotation in 3D Slicer (left, green overlay) and pylidc viewer (right, red outline overlay). The annotation shown corresponds to the largest nodule in the collection (LIDC‐IDRI‐0834 nodule 1). [Color figure can be viewed at wileyonlinelibrary.com]

Consistency of visualization of the segmentations between *pylidc* representation and the DICOM representation was confirmed for a subset of data using *pylidc,* and independently implemented capability of DICOM SEG visualization in 3D Slicer^35^ (version 4.10.2, https://slicer.org) (see Fig. 3) and OHIF Viewer^36^ (version 3.8.13, https://docs.ohif.org/). Further details describing visualization of the DICOM SEG content are discussed in the Usage section.

## 3 DATA FORMAT AND USAGE NOTES

The dataset consists of 6859 DICOM SEG objects (one per annotation of a nodule larger than 3 mm), and 6859 DICOM SR objects that follow TID 1500, containing nine qualitative and three quantitative evaluations of the nodule each (exception to this is subject LIDC‐IDRI‐0510 “Annotation 1708”, which is lacking “Internal structure” assessment). The stored objects follow the DICOM standard, utilizing collection‐specific conventions discussed earlier, and do not use any private attributes to describe the content, though some self‐describing private codes were required.

Due to the use of standard objects, no collection‐specific tools are required to interact with its content. Any tool that implements support of the relevant parts of the DICOM standard can be used. In the following we describe some of the readily available tools that can be utilized to use the content of the dataset, or convert it into alternative representations.

### 3.A Conversion into alternative representations

The data included in the collection was encoded into DICOM representation using *dcmqi* tools.^18^ *dcmqi* can also be used to convert the DICOM representation into other formats that are popular in the research community. For example, DICOM SEG objects can be converted into NRRD, MHD and NIfTI; DICOM SR‐TID1500 objects can be processed to save the encoded evaluations as JSON objects.

### 3.B Programmatic access

A number of toolkits are available to support interpretation of DICOM objects and access to their content at the level of individual attributes. Open source toolkits providing this functionality include DCMTK and Grassroots DICOM (GDCM) (http://gdcm.sourceforge.net/) in C++, *pydicom* in Python (https://github.com/pydicom/pydicom), dcmjs (https://github.com/dcmjs‐org/dcmjs) in JavaScript, and PixelMed DICOM toolkit in Java (https://www.pixelmed.com/dicomtoolkit.html). Programmatic access to the metadata attributes of DICOM SEG objects should be rather straightforward with the basic understanding of the DICOM concepts. Interpretation of the DICOM SR‐TID1500 is somewhat more complicated if done at the level of individual DICOM attributes rather than at the more abstract SR content tree level. The DCMTK *dcmsr* module provides an Application Programming Interface (API) that allows to iterate DICOM SR tree content. However, that API is only available in C++. The PixelMed toolkit also provides a content tree level abstraction, but only in Java. *pydicom* does not provide the abstraction to iterate over the content of the SR tree, but the recently introduced *highdicom* library^37^ (https://highdicom.readthedocs.io/) does provide APIs for reading and writing both DICOM SEG and SR‐TID1500 objects.

If using tools that do not support DICOM SR tree interrogation, another practical approach is to extract the structured content into an alternative representation, such as XML or JSON. The *dsr2xml* command line tool of DCMTK can be used to convert the content of the DICOM SR document into nonstandard *dcmsr‐*specific XML representation. The *tid1500reader* tool from *dcmqi* will store the SR‐TID1500‐specific content into a *dcmqi‐*specific JSON representation. The PixelMed toolkit contains similar tools (XMLRepresentationOfStructuredReportObjectFactory and JSONRepresentationOfStructuredReportObjectFactory). Although the intermediate representations produced by such tools are not standard, they can be generated using publicly available tools from the standard DICOM binary representation, and can simplify programmatic interpretation of those objects in the absence of a more convenient API functionality in languages other than C++ and Java. There is ongoing work to define a standard for a JSON representation of DICOM SR content,^38^ which should simplify access in future.

### 3.C Visualization

**Fig. 4:**
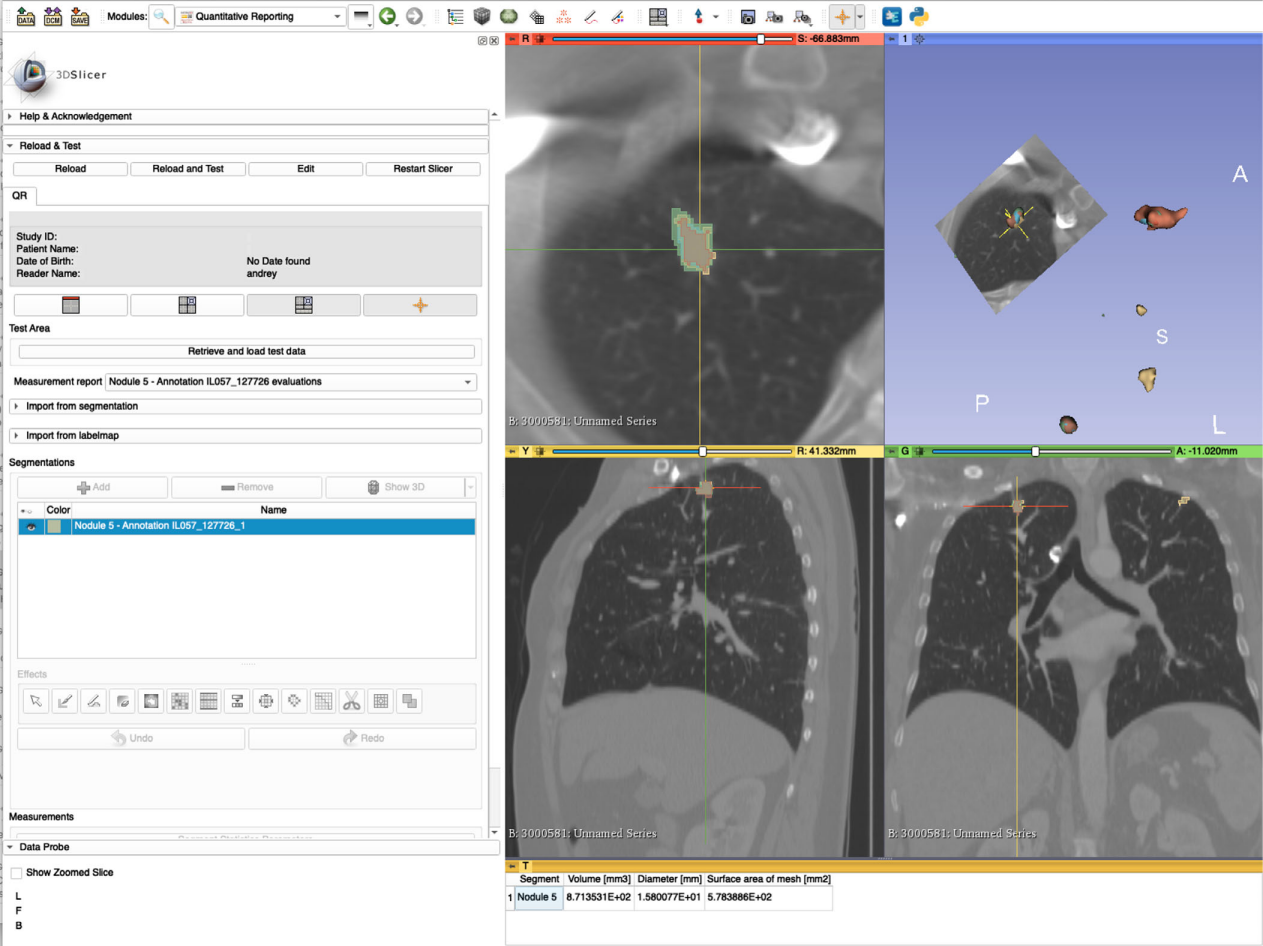
Example of visualization of the annotations and the associated measurements using 3D Slicer QuantitativeReporting extension. Shown is one of the CT scans and the corresponding annotations for subject LIDC‐IDRI‐0055. [Color figure can be viewed at wileyonlinelibrary.com]

Visualization of the DICOM SEG objects and the associated measurements can be performed using the *3D Slicer* software^35^ (https://slicer.org) (see Fig. 4). Visualization tests performed for the dataset utilized the latest stable version of 3D Slicer 4.10.2. The *QuantitativeReporting* extension^18^ (https://github.com/QIICR/QuantitativeReporting) needs to be installed first, since support of DICOM SEG and SR‐TID1500 is not available in the core application. The extension can be installed by first downloading the latest version of the 3D Slicer application package from https://download.slicer.org, and then using the *Extension Manager* to install the extension. Detailed installation instructions are available in the extension documentation. Once installed, DICOM images can be imported into the application using the DICOM *Browser* module, after which any SR object from the collection can be loaded, triggering automatic loading of the corresponding SEG and CT image series.

*OHIF Viewer* is an open source, web‐based, medical imaging viewer^36^ that implements support for DICOM SEG; the support of SR‐TID1500 in the viewer is currently under development. In order to use *OHIF Viewer* for visualization of the DICOM LIDC annotation dataset, its content along with the corresponding DICOM CT images needs to be made available on a DICOM server that implements the standard DICOMweb interface.

Other tools that support visualization and interpretation of DICOM SEG and DICOM SR‐TID1500 objects include the Medical Imaging Toolkit (MITK)^39^ and ePAD,^40^ as well as a number of commercially available platforms.^41^

### 3.D Metadata search and query

Until recently, interrogation of the complete metadata stored in DICOM objects required implementation of a number of steps that could be accomplished using a number of readily available tools, but which required custom setup and maintenance (i.e., extraction and transformation into a representation suitable for a selected database technology). Earlier efforts alleviate some of those steps to some degree, but do not necessarily provide a turn‐key solution. One example of such effort is the Dicoogle platform,^42^ which allows users to “index nearly all metadata and perform free text, keyword‐based, and range‐based queries.”

**Fig. 5:**
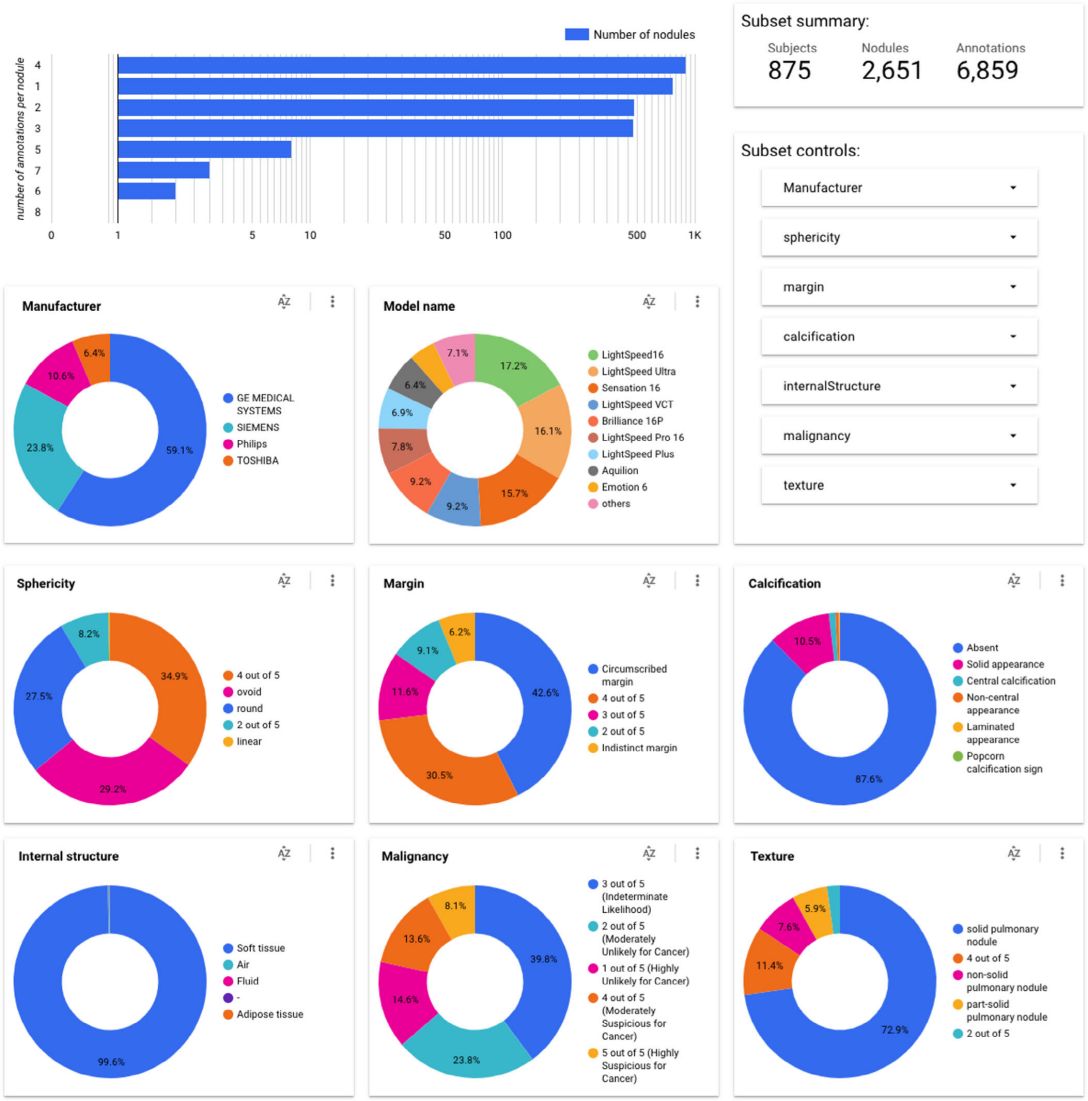
Interactive Google DataStudio dashboard allowing to explore the metadata accompanying the annotations included in the presented collection. Note that the individual categories associated with each pie chart plot are ordered by number of occurrences (e.g., “3 out of 5 (Indeterminate Likelihood)” is the most common value assigned to the “Malignancy” category). The dashboard is publicly available at https://bit.ly/39EaVXT. [Color figure can be viewed at wileyonlinelibrary.com]

**Fig. 6:**
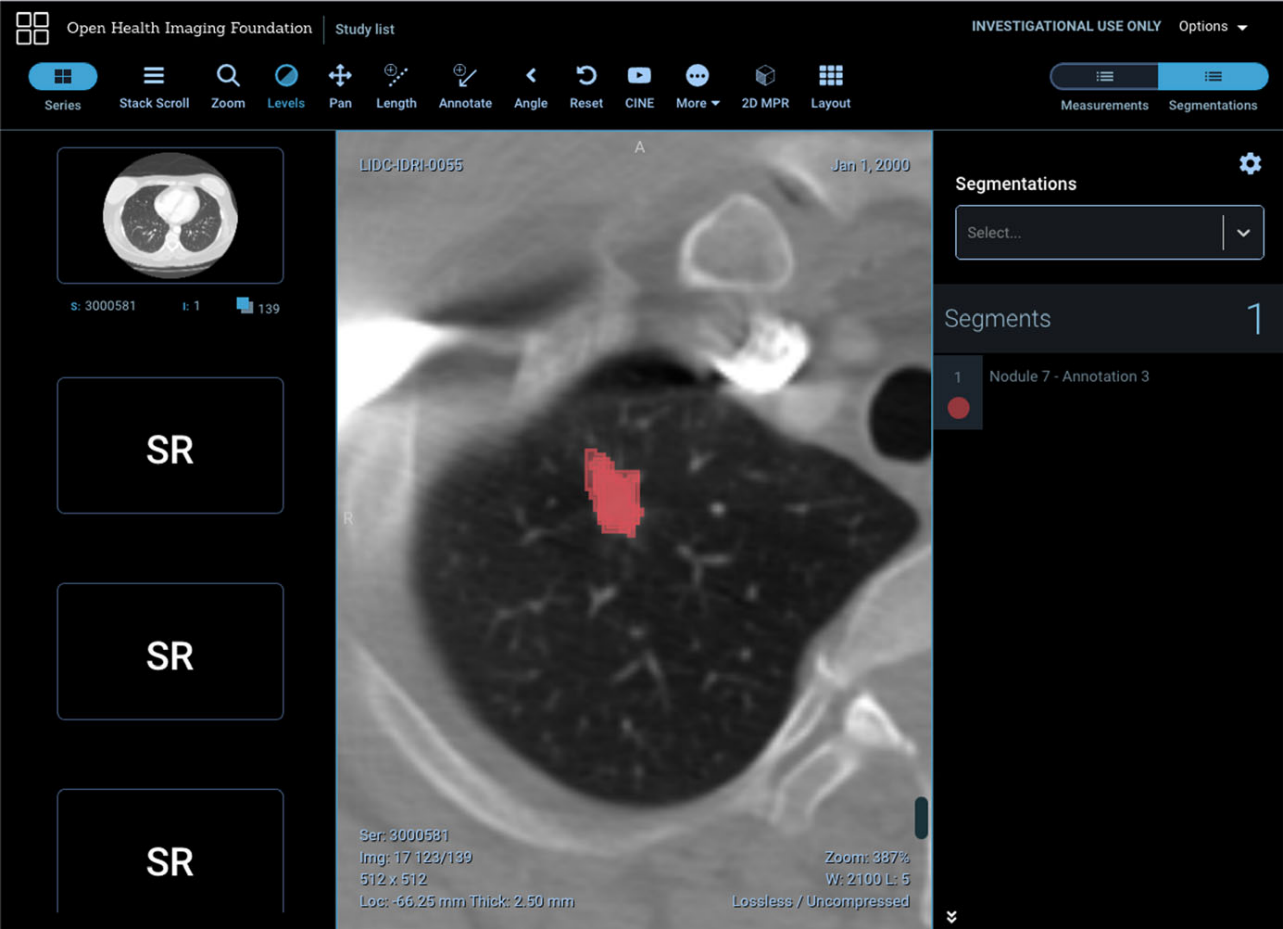
Example of visualization of the annotations and the associated measurements using OHIF Viewer. Shown is one of the CT scans and the corresponding annotations for subject LIDC‐IDRI‐0055. [Color figure can be viewed at wileyonlinelibrary.com]

As part of the work on this dataset, we experimented with the recently introduced Google Healthcare API (GHC),^43^ which is a new component of the Google Cloud Platform implementing the steps above, to a large degree hiding the related complexities. GHC provides a range of tools to support operations on DICOM data that include DICOM data stores that can be interfaced via DICOMweb, and automatic extraction of DICOM metadata into BigQuery tables. Using GHC, we followed the procedures described in GHC documentation^44^ to upload the collection presented in this article into a storage bucket, import it into a DICOM store, and export DICOM metadata into a BigQuery table. We then used the query listed in Appendix S2 to extract the evaluations associated with the annotated lesions from the hierarchy of the SR tree into a table listing all evaluations for an individual, with each annotation corresponding to a single row in the resulting table. The resulting table was used to perform queries validating the content, as discussed in the earlier section. We also created a publicly accessible dashboard that utilizes Google DataStudio to support faceted exploration of the DICOM metadata stored in the BigQuery tables (see Fig. 5, dashboard is accessible at https://bit.ly/39EaVXT). To test interoperability of the collection content hosted in the GHC DICOM store, we configured an instance of open source *OHIF Viewer* ^36^ to interface the GHC DICOM store, and confirmed the generated content can be visualized (see Fig. 6).

## 4 DISCUSSION

Our primary objective in creating this dataset was to make its content more usable and accessible by commonly available tools, and to enable harmonization with other standardized datasets. There have been previous efforts that proposed implementations and platforms to support exploration of the LIDC‐IDRI collection. As part of the original LIDC effort, Meyer et al. described constructing binary nodule masks from the edge maps, computing nodule volume data by summing each radiologist‐method combination’s nodule mask and generating probability maps.^45^ Lin et al. proposed a nodule viewing system developed specifically for the LIDC‐IDRI collection consisting of a custom nodule viewing interface interacting with a MySQL database.^9^ One of the authors (D.C.) previously described conversion of LIDC nodule probability maps produced by the LIDC MAX tool^46^ into DICOM SEG objects and derived measurements into DICOM SR as an intermediate step in generating an image hyperlinked tabular representation for review.^10^ Another custom viewing platform was developed by Zeng et al.^11^ Lampert et al. released a Matlab‐based toolbox for extracting individual annotations from the XML files and converting them, and the DICOM images, into TIFF format for easier processing in Matlab, as part of an effort to use LIDC annotations.^12^ Similarly, Hancock et al. developed a Python‐based package that supports querying of the LIDC annotations and their display using custom user interface as part of their work developing nodule classification system.^13^

We considered only volumetrically annotated nodules ≥ 3 mm, even though the project also generated annotations of non‐nodules ≥ 3 mm and nodules < 3 mm. Those additional annotations were not the primary focus of the database, according to its creators.^5^ Considering those nonvolumetric annotations would require additional efforts to parse and reuse (e.g., as of writing, those categories of annotations are not parsed by *pylidc*), we decided the effort to harmonize them into DICOM is not justified. This, however, could be reconsidered in the future.

Establishing consistency of the DICOM re‐encoded dataset with the original LIDC annotations is not straightforward. Armato et al.^5^ provide nodule‐level summary statistics noting 2669 as the total number of lesions identified by visual inspection of radiologist marks (vs 2651 identified by automatic clustering in *pylidc*). Unfortunately, the original LIDC nodule assignment was not preserved in the publicly shared annotations. Based on the published summaries of the dataset in the LIDC manuscripts, we were not able to locate the total number of annotations for nodules ≥ 3 mm, or the number of subjects that had a nodule ≥ 3 mm.

We followed the approach of developing a standard representation of the data instead of a data‐specific visualization and query tools. As we demonstrated in this paper, such a standard representation allows using off‐the‐shelf components for data exploration. There are numerous advantages to this approach. Existing platforms, such as 3D Slicer and OHIF Viewer, provide comprehensive visualization capabilities, which would be difficult to re‐implement in a custom package. Versatile analysis capabilities available within tools like 3D Slicer make it possible to conduct additional analyses on the data (e.g., radiomics feature extraction or deformable registration) without the need to first convert it into a suitable format.

The use of standard DICOM objects for data representation enables harmonization of the LIDC‐IDRI annotations with other annotations already available in the TCIA collections (e.g., QIN‐HEADNECK,^17^ QIN‐Prostate‐Repeatability^47^ and NSCLC‐Radiomics^48^ are just some of the examples of the collections that utilize the same type of standard objects for storing annotations and derived measurements). The practical implication of this is that the same visualization, query and processing tools can be applied to all those collections. It becomes possible to perform combined queries on the standard content, such as to search for segmentations of a given type based on the standard codes describing the segmentation. It also becomes possible to support interoperability and harmonization with future, as yet unknown software tools and datasets that implement the standard. There is a strong evidence that more and more datasets following this approach will be available in the near future. The Integrating the Healthcare Enterprise (IHE) Technical framework “AI Results” supplement^49^ aims to formalize and encourage the use of standard DICOM objects, including SEG and SR‐TID1500, by the commercial vendors of radiology PACS and viewer platforms, paving the way for clinical workstations to generate results directly usable in other systems implementing the standard.

Many of the components discussed in this article will be available in the National Cancer Institute Imaging Data Commons (IDC) (https://imagingdatacommons.github.io/), a Minimal Viable Product (MVP) of which is currently under development. IDC will utilize the Google Cloud Platform (GCP) and the Google Health Cloud (GHC) API to host and serve up the content of TCIA, co‐located with the various computational and query resources, and to integrate imaging data with the other cloud‐based sources of data. IDC will rely on the standard DICOM representation of images and associated data such as annotations in SEG and SR, the DICOM data model and the standard DICOMweb API to support organization, query, access, visualization and processing of the TCIA content. The presented LIDC annotations dataset will be included in the initial offering of the IDC.

Limitations of the dataset described herein include lack of information on the pathology‐confirmed malignancy of the individual nodules and lack of the information about the reader performing the individual annotation (this would allow evaluation of intra‐ and inter‐reader variability). These limitations are present in the data collected by the LIDC‐IDRI consortium, cannot be remediated, and are not specific to the current effort. We also did not encode information about the annotations for the nodules other than “nodules ≥ 3 mm”. This was done due to lower perceived value of those annotations, and also for expediency purposes, since those annotations were not parsed by the *pylidc* package. In the future, we might consider revisiting this decision, if there is sufficient interest in the community in accessing those annotations.

## 5 CONCLUSION

The dataset contains standard DICOM SEG and SR representations of the annotations collected by the LIDC‐IDRI consortium. These representations improve conformance to the FAIR data principles, and are harmonized with a growing number of other TCIA collections that also contain standard representations for the analysis results. The dataset makes it possible to utilize the growing number of tools that implement the relevant parts of the DICOM standard for the visualization and processing of annotated images.

## Supporting information


**Appendix S1**. Dictionary of the coded terms used in the dataset.

**Appendix S2**. Relevant SQL queries operating on the BigQuery table containing the LIDC annotations collection DICOM metadata.
