# Supplementary material for: DICOM re‐encoding of volumetrically annotated Lung Imaging Database Consortium (LIDC) nodules
Source: Med Phys. 2020 Sep 6;47(11):5953–65. doi: 10.1002/mp.14445 (PMC7721965; doi:10.1002/mp.14445)
Supplement: Supplementary file 1 — Appendix S1. Dictionary of the coded terms used in the dataset. [file MP-47-5953-s001.doc]

Appendix S-1: Dictionary of the coded terms used in the dataset

| **Table 1. LIDC-IDRI Evaluation Concepts** |
| --- |

| **Coding Scheme Designator** | **Code Value** | **Code Meaning** | **LIDC-IDRI concept name verbatim** | **LIDC definition** |
| --- | --- | --- | --- | --- |
| NCIt | [C45992](http://bioportal.bioontology.org/ontologies/NCIT?p=classes&conceptid=http%3A%2F%2Fncicb.nci.nih.gov%2Fxml%2Fowl%2FEVS%2FThesaurus.owl%23C45992) | Subtlety Score | Subtlety | Radiologist assessment of nodule subtlety on 1-5 scale |
| 99LIDCQIICR | 200 | Internal structure | Internal structure | Radiologist assessment of nodule internal structure |
| NCIt | [C3672](http://bioportal.bioontology.org/ontologies/NCIT?p=classes&conceptid=http%3A%2F%2Fncicb.nci.nih.gov%2Fxml%2Fowl%2FEVS%2FThesaurus.owl%23C3672) | Calcification | Calcification | Radiologist assessment of internal calcification of nodule |
| 99LIDCQIICR | 400 | Sphericity | Sphericity | Radiologist assessment of shape of nodule in terms of its roundness/sphericity with only 3 terms defined |
| NCIt | [C25563](http://bioportal.bioontology.org/ontologies/NCIT/?p=classes&conceptid=http%3A%2F%2Fncicb.nci.nih.gov%2Fxml%2Fowl%2FEVS%2FThesaurus.owl%23C25563&jump_to_nav=true) | Margin | Margin | Radiologist assessment of nodule margin on a 1-5 scale with only the extreme values explicitly defined |
| NCIt | [C62175](http://purl.obolibrary.org/obo/NCIT_C62175) | Lobular Pattern | Lobulation | Radiologist assessment of nodule lobulation on a 1-5 scale with only the extreme values explicitly defined |
| NCIt | [C28749](http://bioportal.bioontology.org/ontologies/NCIT/?p=classes&conceptid=http%3A%2F%2Fncicb.nci.nih.gov%2Fxml%2Fowl%2FEVS%2FThesaurus.owl%23C28749&jump_to_nav=true) | Spiculate (synonym: Spiculation) | Spiculation | Radiologist assessment of nodule spiculation on a 1-5 scale with only the extreme values explicitly defined |
| NCIt | [C41144](http://bioportal.bioontology.org/ontologies/NCIT/?p=classes&conceptid=http%3A%2F%2Fncicb.nci.nih.gov%2Fxml%2Fowl%2FEVS%2FThesaurus.owl%23C41144&jump_to_nav=true) | Texture | Texture | Radiologist assessment of nodule internal texture with only 3 terms defined |
| RadLex | [RID36042](http://www.radlex.org/RID/#RID36042) | Malignant neoplasm (synonym: Malignancy) | Likelihood of malignancy | Radiologist subjective assessment of likelihood of malignancy of this nodule (ASSUMING 60-year-old male smoker) |

| **Table 2. LIDC-IDRI Evaluation Concept Values.** |
| --- |

| **Coding Scheme Designator** | **Code Value** | **Code Meaning** | **LIDC-IDRI concept name verbatim** |
| --- | --- | --- | --- |
| ***Subtlety score^^[[1]](#footnote-0)^^*** | | | |
| 99LIDCQIICR | 101 | 1 out of 5 (Extremely subtle) | 1 - Extremely subtle |
| 99LIDCQIICR | 102 | 2 out of 5 (Moderately subtle) | 2 - Moderately subtle |
| 99LIDCQIICR | 103 | 3 out of 5 (Fairly subtle) | 3 - Fairly subtle |
| 99LIDCQIICR | 104 | 4 out of 5 (Moderately obvious) | 4 - Moderately obvious |
| 99LIDCQIICR | 105 | 5 out of 5 (Obvious) | 5 - Obvious |
| ***Internal structure*** | | | |
| NCIt | [C12471](http://bioportal.bioontology.org/ontologies/NCIT/?p=classes&conceptid=http%3A%2F%2Fncicb.nci.nih.gov%2Fxml%2Fowl%2FEVS%2FThesaurus.owl%23C12471&jump_to_nav=true) | Soft tissue | Soft tissue |
| NCIt | [C25278](http://bioportal.bioontology.org/ontologies/NCIT/?p=classes&conceptid=http%3A%2F%2Fncicb.nci.nih.gov%2Fxml%2Fowl%2FEVS%2FThesaurus.owl%23C25278&jump_to_nav=true) | Fluid | Fluid |
| NCIt | [C12472](http://bioportal.bioontology.org/ontologies/NCIT/?p=classes&conceptid=http%3A%2F%2Fncicb.nci.nih.gov%2Fxml%2Fowl%2FEVS%2FThesaurus.owl%23C12472&jump_to_nav=true) | Adipose tissue | Fat |
| NCIt | [C73434](http://bioportal.bioontology.org/ontologies/NCIT/?p=classes&conceptid=http%3A%2F%2Fncicb.nci.nih.gov%2Fxml%2Fowl%2FEVS%2FThesaurus.owl%23C73434&jump_to_nav=true) | Air | Air |
| ***Calcification*** | | | |
| RadLex | [RID35453](http://www.radlex.org/RID/#RID35453) | Popcorn calcification sign | 1 - Popcorn appearance |
| 99LIDCQIICR | 302 | Laminated appearance | 2 - Laminated appearance |
| RadLex | [RID5741](http://www.radlex.org/RID/RID5741) | Solid | 3 - Solid appearance |
| 99LIDCQIICR | 304 | Non-central appearance | 4 - Non-central appearance |
| RadLex | [RID5827](http://www.radlex.org/RID/RID5827) | Central | 5 - Central calcification |
| RadLex | [RID28473](http://www.radlex.org/RID/#RID28473) | Absent | 6 - Absent |
| ***Sphericity*** | | | |
| RadLex | [RID5811](http://www.radlex.org/RID/#RID5811) | linear | 1 - Linear appearance |
| 99LIDCQIICR | 002 | 2 out of 5 | 2 |
| RadLex | [RID5800](http://www.radlex.org/RID/#RID5800) | ovoid | 3 - Ovoid appearance |
| 99LIDCQIICR | 004 | 4 out of 5 | 4 |
| RadLex | [RID5799](http://www.radlex.org/RID/#RID5799) | round | 5 - Round appearance |
| ***Margin*** | | | |
| RadLex | [RID5709](http://www.radlex.org/RID/#RID5709) | Indistinct margin (synonym: poorly defined margin) | 1 - Poorly defined |
| 99LIDCQIICR | 002 | 2 out of 5 | 2 |
| 99LIDCQIICR | 003 | 3 out of 5 | 3 |
| 99LIDCQIICR | 004 | 4 out of 5 | 4 |
| RadLex | [RID5707](http://www.radlex.org/RID/#RID5707) | Circumscribed margin (synonym: sharpy-defined margin) | 5 - Sharp margin |
| ***Lobular Pattern*** | | | |
| 99LIDCQIICR | 601 | 1 out of 5 (No lobulation) | 1 - No lobulation |
| 99LIDCQIICR | 002 | 2 out of 5 | 2 |
| 99LIDCQIICR | 003 | 3 out of 5 | 3 |
| 99LIDCQIICR | 004 | 4 out of 5 | 4 |
| 99LIDCQIICR | 605 | 5 out of 5 (Marked lobulation) | 5 - Marked lobulation |
| ***Spiculation*** | | | |
| 99LIDCQIICR | 701 | 1 out of 5 (No spiculation) | 1 - No spiculation |
| 99LIDCQIICR | 002 | 2 out of 5 | 2 |
| 99LIDCQIICR | 003 | 3 out of 5 | 3 |
| 99LIDCQIICR | 004 | 4 out of 5 | 4 |
| 99LIDCQIICR | 705 | 5 out of 5 (Marked spiculation) | 5 - Marked spiculation |
| ***Texture*** | | | |
| RadLex | [RID50153](http://www.radlex.org/RID/#RID50153) | Non-solid pulmonary nodule (synonym: pure ground-glass pulmonary nodule) | 1 - Non-solid/Ground Glass Opacity |
| 99LIDCQIICR | 002 | 2 out of 5 | 2 |
| RadLex | [RID50152](http://www.radlex.org/RID/#RID50152) | part-solid pulmonary nodule | 3 - Part-solid/mixed |
| 99LIDCQIICR | 004 | 4 out of 5 | 4 |
| RadLex | [RID50151](http://www.radlex.org/RID/#RID50151) | solid pulmonary nodule | 5 - Solid texture |
| ***Malignancy***^^[[2]](#footnote-1)^^ | | | |
| 99LIDCQIICR | 901 | 1 out of 5 (Highly Unlikely for Cancer) | 1 - Highly Unlikely for Cancer |
| 99LIDCQIICR | 902 | 2 out of 5 (Moderately Unlikely for Cancer) | 2 - Moderately Unlikely for Cancer |
| 99LIDCQIICR | 903 | 3 out of 5 (Indeterminate Likelihood) | 3 - Indeterminate Likelihood |
| 99LIDCQIICR | 904 | 4 out of 5 (Moderately Suspicious for Cancer) | 4 - Moderately Suspicious for Cancer |
| 99LIDCQIICR | 905 | 5 out of 5 (Highly Suspicious for Cancer) | 5 - Highly Suspicious for Cancer |

#

# References

1. [McNitt-Gray MF, Armato SG III, Meyer CR, et al. The Lung Image Database Consortium (LIDC) Data Collection Process for Nodule Detection and Annotation. *Acad Radiol*. 2007;14(12):1464-1474. doi:](http://paperpile.com/b/sev7KD/rPz6t)[10.1016/j.acra.2007.07.021](http://dx.doi.org/10.1016/j.acra.2007.07.021)

1. Annotated XML file contains definitions for the subtlety score only for scores 1 and 5, while Fig.7 showing software interface in [^1^](https://paperpile.com/c/sev7KD/rPz6t) contains definitions for all of the scores, as listed in the table. [↑](#footnote-ref-0)
2. Note minor inconsistencies in the definitions in the annotated XML file as compared to Fig.7 in McNitt-Gray et al.[^1^](https://paperpile.com/c/sev7KD/rPz6t). [↑](#footnote-ref-1)
