# Supplementary material for: DICOM re‐encoding of volumetrically annotated Lung Imaging Database Consortium (LIDC) nodules
Source: Med Phys. 2020 Sep 6;47(11):5953–65. doi: 10.1002/mp.14445 (PMC7721965; doi:10.1002/mp.14445)
Supplement: Supplementary file 2 — Appendix S2. Relevant SQL queries operating on the BigQuery table containing the LIDC annotations collection DICOM metadata. [file MP-47-5953-s002.doc]

Appendix S-2: Relevant SQL queries operating on the BigQuery table containing the LIDC annotations collection DICOM metadata

Note that the table name is a placeholder and will need to be replaced with the table accessible by the user executing the query. Standard capabilities of Google Healthcare can be used to construct such table from the DICOM content presented in this manuscript.

The queries below are intended to serve merely as an example of how the relevant attributes can be extracted from the DICOM BigQuery metadata tables.

These queries are also available at <https://github.com/QIICR/lidc2dicom>.

# Extraction of annotation evaluations

WITH

measurementGroups AS (

WITH

level2ContentItems AS (

WITH

structuredReports AS (

SELECT

PatientID,

SOPInstanceUID,

SeriesDescription,

references.StudyInstanceUID AS referencedStudyInstanceUID,

ContentSequence

FROM

**<TABLE NAME>**

CROSS JOIN

UNNEST(CurrentRequestedProcedureEvidenceSequence) AS references

WHERE

Modality = "SR")

SELECT

PatientID,

SOPInstanceUID,

SeriesDescription,

referencedStudyInstanceUID,

contentItems

FROM

structuredReports

CROSS JOIN

UNNEST(ContentSequence) AS contentItems )

SELECT

PatientID,

SOPInstanceUID,

SeriesDescription,

referencedStudyInstanceUID,

contentSequence

FROM

level2ContentItems

CROSS JOIN

UNNEST (contentItems.ContentSequence) AS contentSequence

WHERE

contentSequence.ValueType = "CONTAINER"

AND contentSequence.ConceptNameCodeSequence[

OFFSET

(0)].CodeMeaning = "Measurement Group" ),

quantitativeMeasurements AS (

WITH

numItems AS(

SELECT

PatientID,

SOPInstanceUID,

SeriesDescription,

referencedStudyInstanceUID,

contentSequence.ConceptNameCodeSequence[

OFFSET

(0)] AS ConceptNameCodeSequence,

contentSequence.MeasuredValueSequence[

OFFSET

(0)] AS MeasuredValueSequence,

contentSequence.MeasuredValueSequence[

OFFSET

(0)].MeasurementUnitsCodeSequence[

OFFSET

(0)] AS MeasurementUnits

FROM

measurementGroups

CROSS JOIN

UNNEST (contentSequence.ContentSequence) AS contentSequence

WHERE

contentSequence.ValueType = "NUM"),

trackingIdentifier AS (

SELECT

SOPInstanceUID,

contentSequence.TextValue AS trackingIdentifier

FROM

measurementGroups

CROSS JOIN

UNNEST (contentSequence.ContentSequence) AS contentSequence

WHERE

contentSequence.ValueType = "TEXT"

AND contentSequence.ConceptNameCodeSequence[

OFFSET

(0)].CodeMeaning = "Tracking Identifier" )

SELECT

PatientID,

SOPInstanceUID,

SeriesDescription,

referencedStudyInstanceUID,

SUM(CASE

WHEN ConceptNameCodeSequence.CodeMeaning = "Volume" THEN SAFE_CAST(MeasuredValueSequence.NumericValue[ OFFSET (0)] AS NUMERIC)

ELSE

0

END

) AS volume,

SUM(CASE

WHEN ConceptNameCodeSequence.CodeMeaning = "Surface area of mesh" THEN SAFE_CAST(MeasuredValueSequence.NumericValue[ OFFSET (0)] AS numeric)

ELSE

0

END

) AS surface,

SUM(CASE

WHEN ConceptNameCodeSequence.CodeMeaning = "Diameter" THEN SAFE_CAST(MeasuredValueSequence.NumericValue[ OFFSET (0)] AS numeric)

ELSE

0

END

) AS diameter

FROM

numItems

GROUP BY

1,

2,

3,

4),

qualitativeMeasurements AS (

WITH

codeItems AS(

SELECT

PatientID,

SOPInstanceUID,

SeriesDescription,

referencedStudyInstanceUID,

contentSequence.ConceptNameCodeSequence[

OFFSET

(0)] AS ConceptNameCodeSequence,

contentSequence.ConceptCodeSequence[

OFFSET

(0)] AS ConceptCodeSequence

FROM

measurementGroups

CROSS JOIN

UNNEST (contentSequence.ContentSequence) AS contentSequence

WHERE

contentSequence.ValueType = "CODE")

SELECT

PatientID,

SOPInstanceUID,

SeriesDescription,

referencedStudyInstanceUID,

MAX(CASE

WHEN ConceptNameCodeSequence.CodeMeaning = "Subtlety score" THEN ConceptCodeSequence.CodeMeaning

ELSE

"-"

END

) AS subtlety,

MAX(CASE

WHEN ConceptNameCodeSequence.CodeMeaning = "Internal structure" THEN ConceptCodeSequence.CodeMeaning

ELSE

"-"

END

) AS internal_structure,

MAX(CASE

WHEN ConceptNameCodeSequence.CodeMeaning = "Calcification" THEN ConceptCodeSequence.CodeMeaning

ELSE

"-"

END

) AS calcification,

MAX(CASE

WHEN ConceptNameCodeSequence.CodeMeaning = "Sphericity" THEN ConceptCodeSequence.CodeMeaning

ELSE

"-"

END

) AS sphericity,

MAX(CASE

WHEN ConceptNameCodeSequence.CodeMeaning = "Margin" THEN ConceptCodeSequence.CodeMeaning

ELSE

"-"

END

) AS margin,

MAX(CASE

WHEN ConceptNameCodeSequence.CodeMeaning = "Lobular Pattern" THEN ConceptCodeSequence.CodeMeaning

ELSE

"-"

END

) AS lobulation,

MAX(CASE

WHEN ConceptNameCodeSequence.CodeMeaning = "Spiculation" THEN ConceptCodeSequence.CodeMeaning

ELSE

"-"

END

) AS spiculation,

MAX(CASE

WHEN ConceptNameCodeSequence.CodeMeaning = "Texture" THEN ConceptCodeSequence.CodeMeaning

ELSE

"-"

END

) AS texture,

MAX(CASE

WHEN ConceptNameCodeSequence.CodeMeaning = "Malignancy" THEN ConceptCodeSequence.CodeMeaning

ELSE

"-"

END

) AS malignancy

FROM

codeItems

GROUP BY

1,

2,

3,

4),

quantWithTracking AS (

WITH

tracking AS (

WITH

trackingIdentifiers AS (

SELECT

SOPInstanceUID,

PatientID,

contentSequence.TextValue AS trackingIdentifier

FROM

measurementGroups

CROSS JOIN

UNNEST (contentSequence.ContentSequence) AS contentSequence

WHERE

contentSequence.ValueType = "TEXT"

AND contentSequence.ConceptNameCodeSequence[

OFFSET

(0)].CodeMeaning = "Tracking Identifier"),

trackingUIDs AS (

SELECT

SOPInstanceUID,

PatientID,

contentSequence.UID AS trackingUID

FROM

measurementGroups

CROSS JOIN

UNNEST (contentSequence.ContentSequence) AS contentSequence

WHERE

contentSequence.ValueType = "UIDREF"

AND contentSequence.ConceptNameCodeSequence[

OFFSET

(0)].CodeMeaning = "Tracking Unique Identifier")

SELECT

trackingIdentifiers.SOPInstanceUID,

trackingIdentifiers.trackingIdentifier,

trackingUIDs.trackingUID

FROM

trackingIdentifiers

JOIN

trackingUIDs

ON

trackingIdentifiers.SOPInstanceUID = trackingUIDs.SOPInstanceUID )

SELECT

tracking.SOPInstanceUID,

tracking.trackingIdentifier,

tracking.trackingUID,

quantitativeMeasurements.diameter,

quantitativeMeasurements.volume,

quantitativeMeasurements.surface

FROM

tracking

JOIN

quantitativeMeasurements

ON

tracking.SOPInstanceUID = quantitativeMeasurements.SOPInstanceUID)

SELECT

qual.PatientID,

qual.SOPInstanceUID,

qual.SeriesDescription,

qual.referencedStudyInstanceUID,

qual.subtlety,

qual.internal_structure AS internalStructure,

qual.sphericity,

qual.margin,

qual.lobulation,

qual.spiculation,

qual.texture,

qual.malignancy,

qual.calcification,

quantWithTracking.diameter,

quantWithTracking.volume,

quantWithTracking.surface,

quantWithTracking.trackingIdentifier,

quantWithTracking.trackingUID

FROM

quantWithTracking

JOIN

qualitativeMeasurements AS qual

ON

qual.SOPInstanceUID = quantWithTracking.SOPInstanceUID

ORDER BY

PatientID

# Collecting number of annotations per nodule

WITH

annotation_counts AS (

SELECT

SegmentSequence[

OFFSET

(0)].TrackingUID AS noduleUID,

COUNT(SegmentSequence[

OFFSET

(0)].TrackingUID) AS annotation_cnt

FROM

**<TABLE NAME>**

WHERE

Modality = "SEG"

GROUP BY

noduleUID

ORDER BY

annotation_cnt DESC),

segmentations_only AS (

SELECT

*

FROM

`idc-tcia.lidc_idri_seg_sr.lidc_idri_seg_sr`

WHERE

Modality = "SEG")

SELECT

DISTINCT(noduleUID),

annotation_cnt,

PatientID,

StudyInstanceUID

FROM

annotation_counts

JOIN

segmentations_only AS full_table

ON

annotation_counts.noduleUID = full_table.SegmentSequence[

OFFSET

(0)].TrackingUID

ORDER BY

annotation_cnt DESC
